# Supplementary material for: Cargo Recognition Mechanisms of Yeast Myo2 Revealed by AlphaFold2-Powered Protein Complex Prediction
Source: Biomolecules. 2022 Jul 26;12(8):1032. doi: 10.3390/biom12081032 (PMC9330073; doi:10.3390/biom12081032)
Supplement: Supplementary file 1 [file biomolecules-12-01032-s001.zip › biomolecules-1806097-Supplymentary-proof.pdf]

Supporting information for

Cargo Recognition Mechanisms of Yeast Myo2 Revealed by AlphaFold2-Powered Protein Complex Prediction

Yong Liu[1,2,3], Lingxuan Li[2,3], Cong Yu[2,4], Fuxing Zeng[2], Fengfeng Niu[2,3,*] and Zhiyi Wei[2,3,*]

[1] SUSTech-HIT Joint PhD Program, Harbin Institute of Technology, Harbin, China 150001; 11849501@mail.sustech.edu.cn
[2] Department of Biology, School of Life Sciences, Southern University of Science and Technology, Shenzhen, China 518055; 12032126@mail.sustech.edu.cn; yuc@sustech.edu.cn; zengfx@sustech.edu.cn
[3] Brain Research Center, School of Life Sciences, Southern University of Science and Technology, Shenzhen, China 518055.
[4] Guangdong Provincial Key Laboratory of Cell Microenvironment and Disease Research, and Shenzhen Key Laboratory of Cell Microenvironment, Shenzhen, Guangdong, China 518055.
[*] Correspondence: weizy@sustech.edu.cn and niuff@sustech.edu.cn

This file includes Supplemental Figures S1–S7 and Table S1.

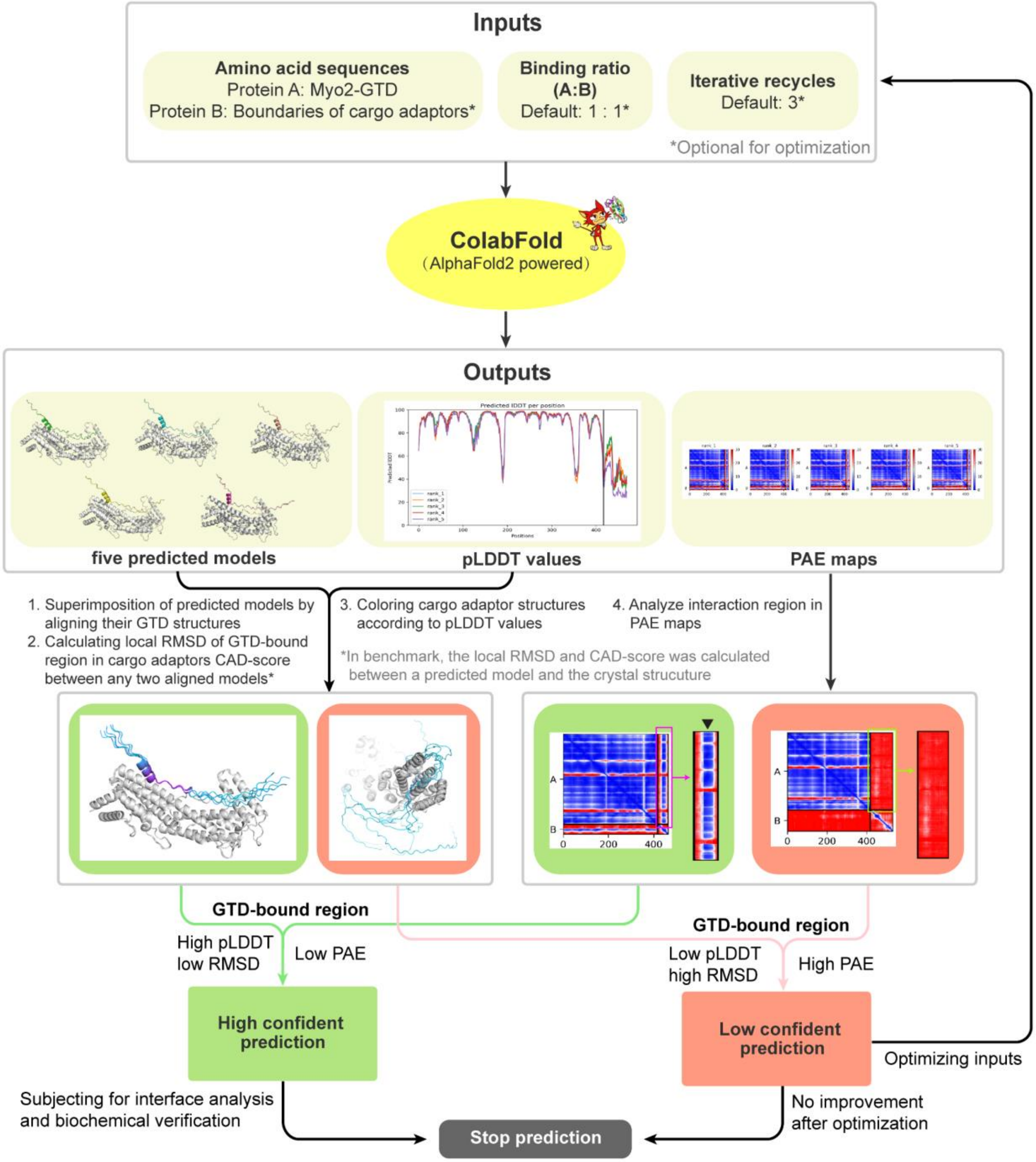

Figure S1. A flow chart for the prediction of Myo2-GTD in complex with cargo adaptors.

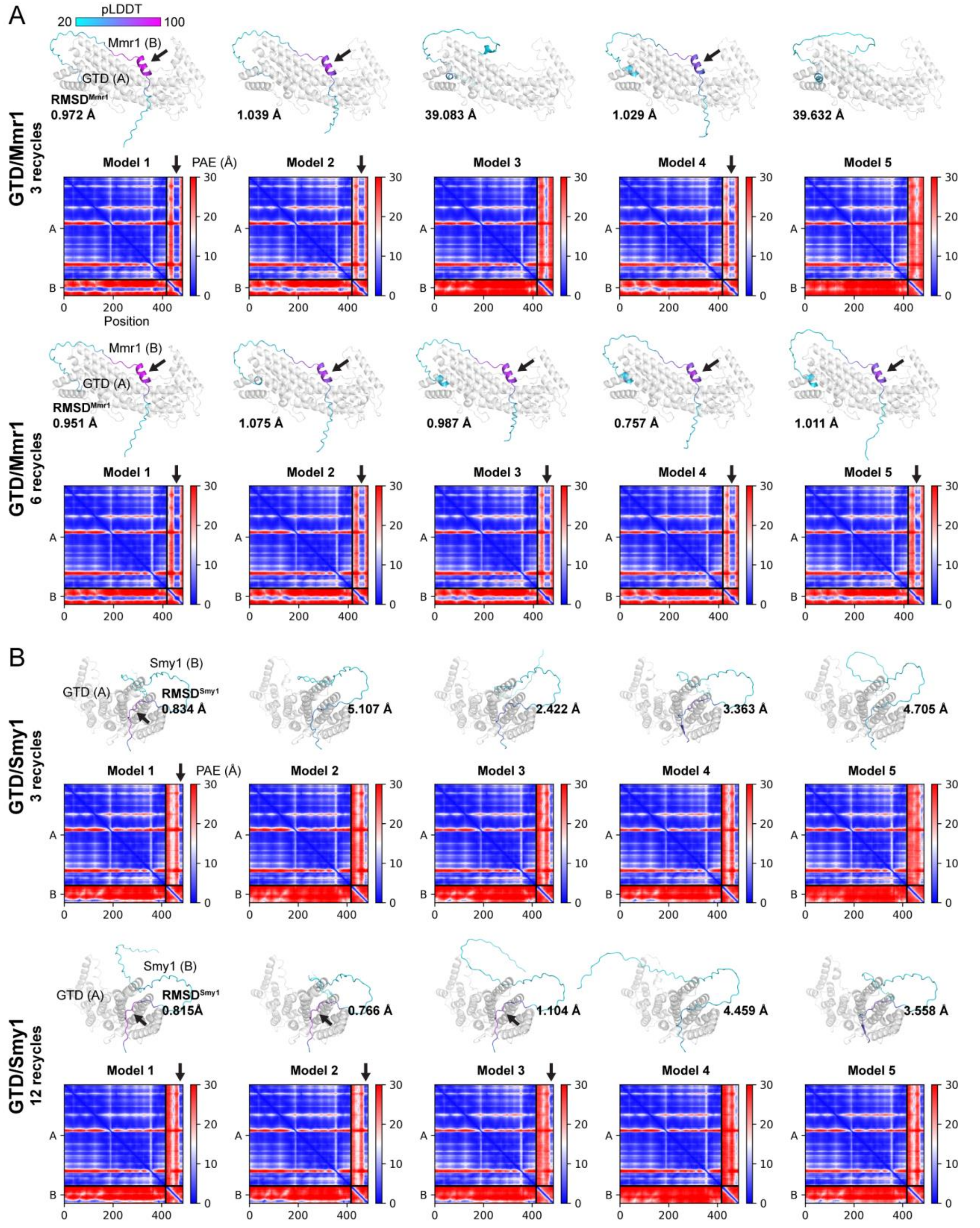

Figure S2. Structure prediction results of Myo2-GTD in complex with Mmr1 or Smy1.**(A and B)** Layouts of the five predicted structures and the corresponding PAE maps of Myo2-GTD in complex with Mmr1-MIS **(A)** or Smy1-MIS

(B) with the calculation of indicated iterative recycles. The MIS regions were colored according to the pLDDT value of each residue. The local RMSD values of the GTD-binding regions between each predicted model and the crystal structure was calculated. The GTD-binding regions in the conserved models and PAE maps were indicated by black arrows.

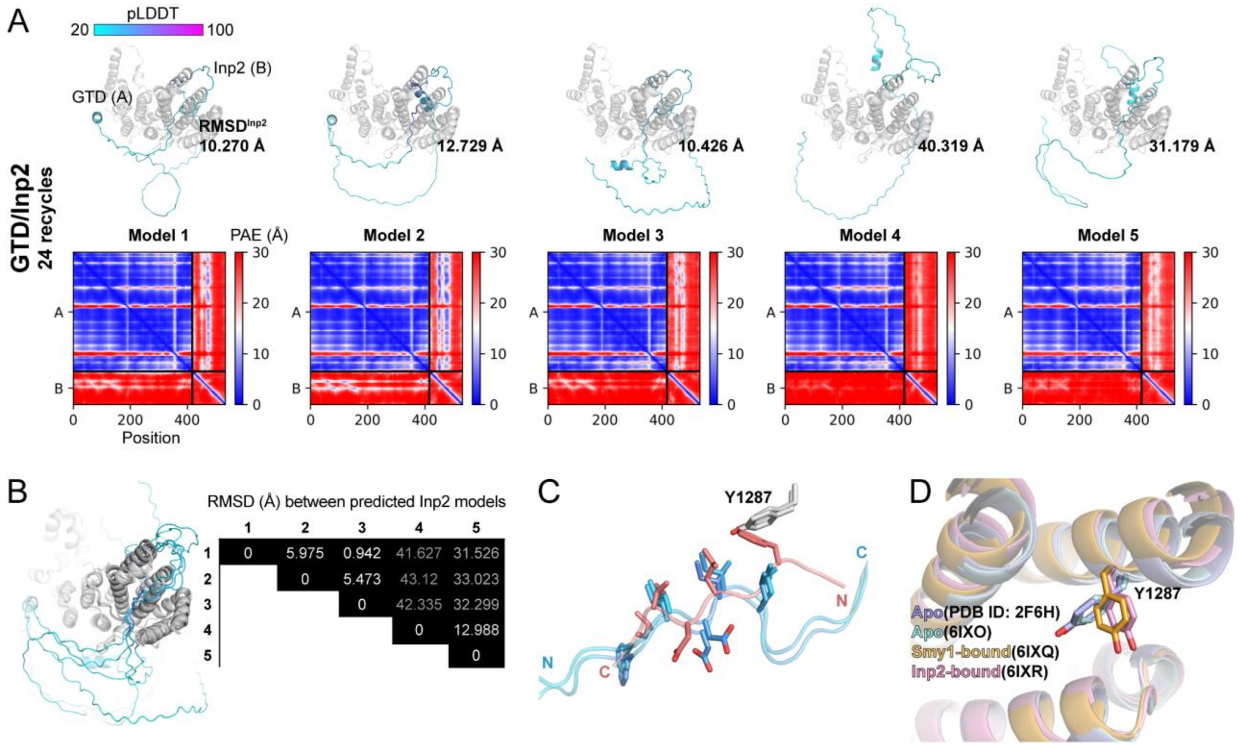

Figure S3. Structure prediction results of the Myo2-GTD in complex with Inp2.**(A)** A layout of the five predicted structures and the corresponding PAE maps of Myo2-GTD in complex with Inp2-MIS with the calculation of 24 recycles. The local RMSD values of the GTD-binding regions between each predicted model and the crystal structure was labeled. **(B)** The four predicted complex structures. The structures were superimposed by aligning their Myo2-GTD structures and Inp2-MIS were colored according to the pLDDT value of each residue. The local RMSD values between any two predicted GTD-binding regions were calculated. **(C)** Comparison of the interface residues of Inp2-MIS between two predicted models and crystal structure. Inp2-MIS has reversed directions in the predicted and crystal structures. Y1287^{GTD} in the predicted models clashes into Inp2-MIS from the crystal structure that has the correct direction. **(D)** Structural comparison showing the different sidechain orientations of Y1287 in the apo and adaptor-bound Myo2-GTD structures.

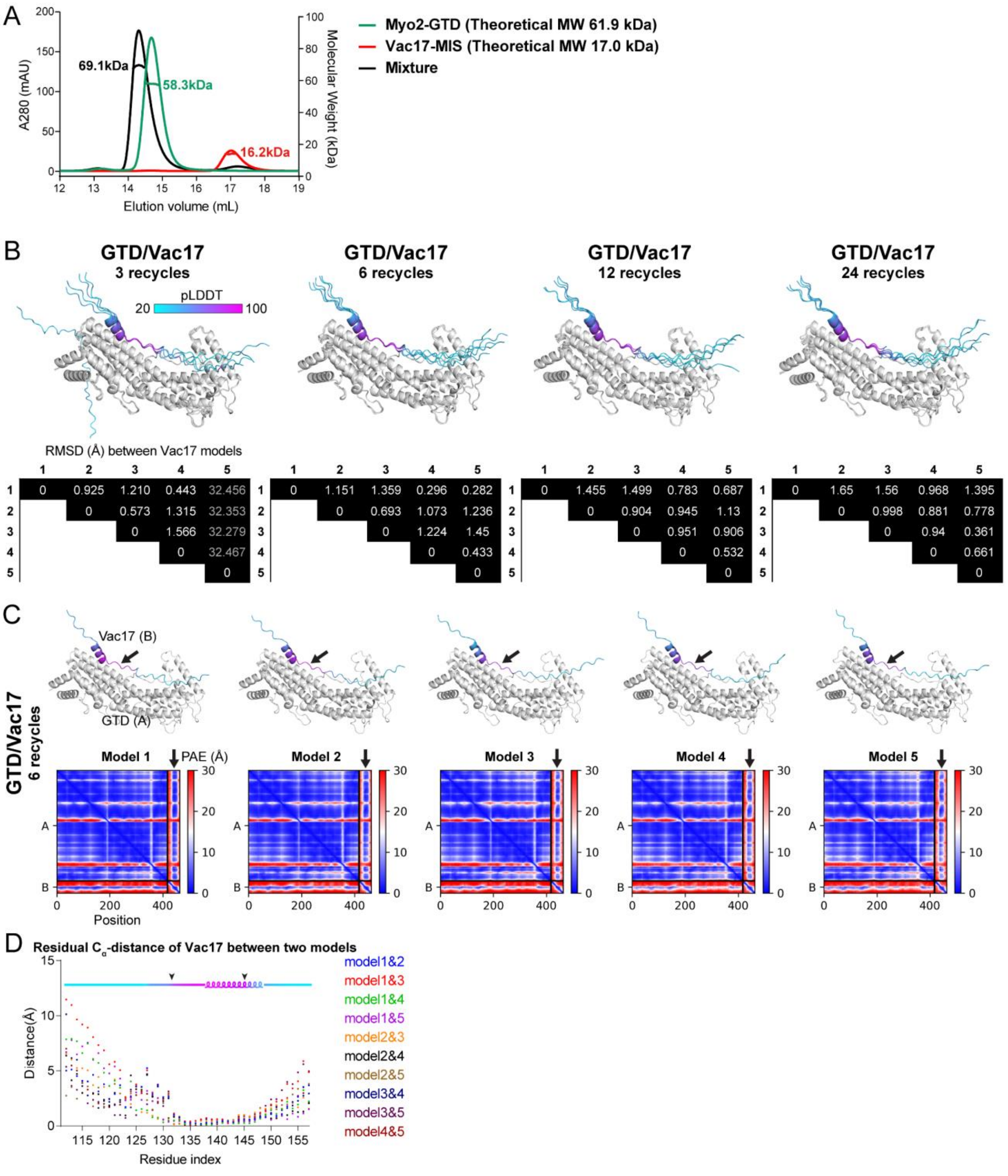

Figure S4. Structure prediction results of the Myo2-GTD in complex with Vac17.**(A)** aSEC profiles of Myo2-GTD, Vac17-MIS and their mixture with the theoretical and experimental molecular weights indicated. **(B)** A layout of the GTD-aligned predicted structures of the Myo2-GTD/Vac17-MIS complex with the calculation of indicated iterative recycles. The local RMSD values between any two predicted GTD-binding regions were calculated. **(C)** A layout of the five predicted structures and the corresponding PAE maps of Myo2-GTD in complex with Vac17-MIS with 6-

recycle calculation. **(D)** Distance plot of the $C\alpha$ atom per residue in Vac17-MIS between each two predicted models. The boundary used for local RMSD calculation is indicated by two arrowheads above the pLDDT-colored secondary structure elements.

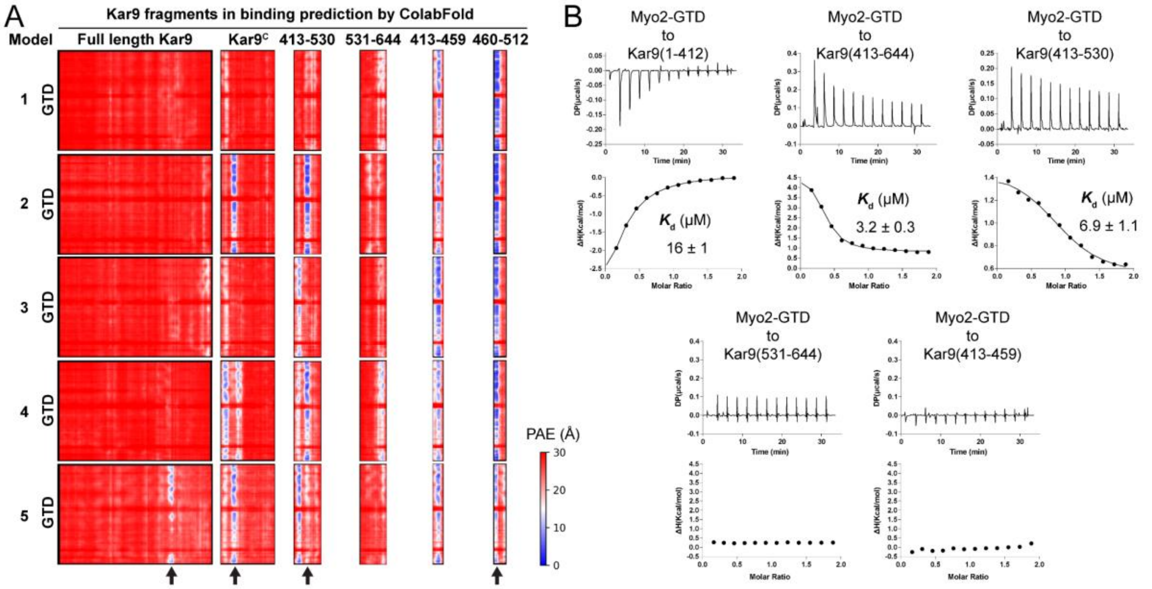

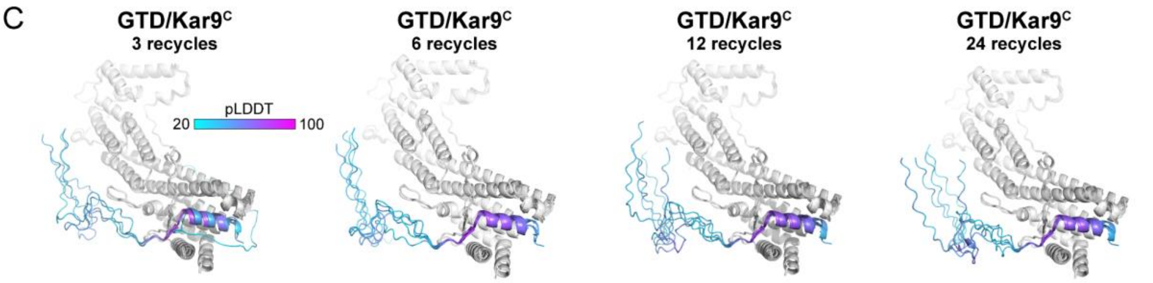

RMSD (Å) between Kar9 models

3 recycles

	1	2	3	4	5
1	0	0.553	0.467	0.46	6.469
2		0	0.893	0.656	6.597
3			0	0.523	6.432
4				0	6.63
5					0

6 recycles

	1	2	3	4	5
1	0	0.557	0.685	0.559	0.559
2		0	0.998	0.795	0.841
3			0	0.377	0.578
4				0	0.436
5					0

12 recycles

	1	2	3	4	5
1	0	0.778	0.721	0.551	0.563
2		0	1.134	0.82	1.058
3			0	0.601	0.589
4				0	0.381
5					0

24 recycles

	1	2	3	4	5
1	0	0.827	0.662	0.524	0.473
2		0	1.218	0.895	1.041
3			0	0.652	0.596
4				0	0.405
5					0

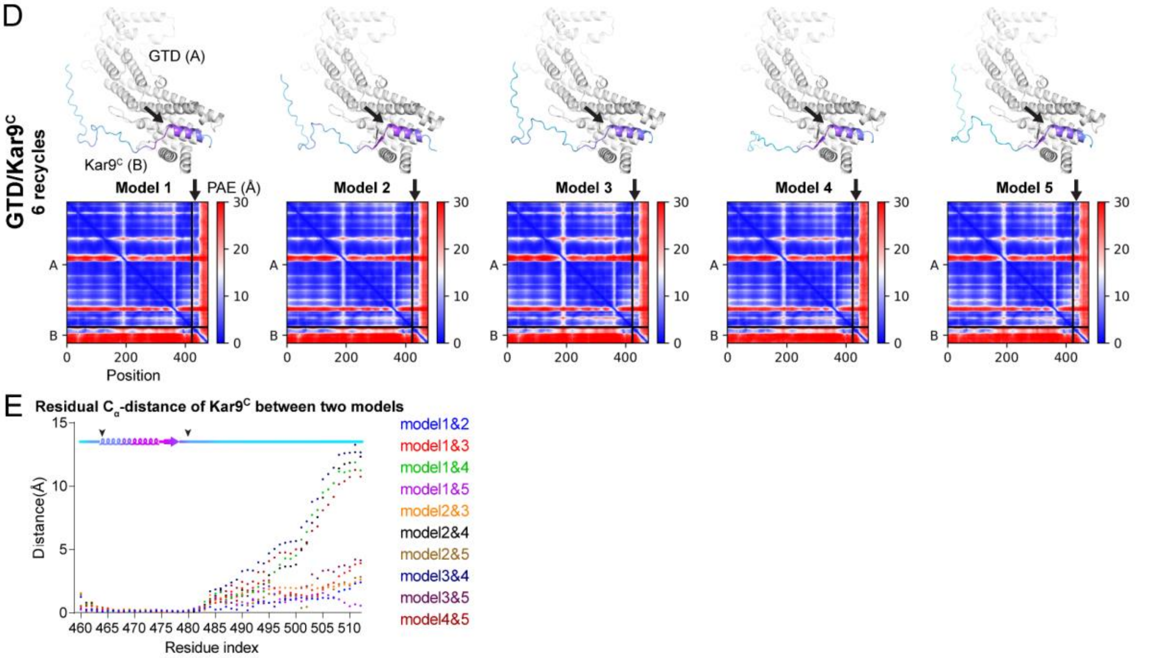

Figure S5. Structure prediction results of the Myo2-GTD in complex with Kar9^C.**(A)** The computational identification of the MIS region in Kar9^C by ColabFold. The intermolecular PAE maps of predicted models generated by inputting the sequences of Myo2-GTD and indicated Kar9 boundaries into ColabFold for the default 3-recycle calculation. The biochemically confirmed GTD-binding region in the Kar9 boundaries were indicated by black arrows. **(B)** ITC-based analysis of the binding of Myo2-GTD to different Kar9 boundaries. **(C)** A layout of the GTD-aligned predicted structures of the Myo2-GTD/Kar9^C-MIS complex. The local RMSD values between any two predicted GTD-binding regions were calculated. **(D)** A layout of the five predicted structures and the corresponding PAE maps of Myo2-GTD in complex with Kar9^C-MIS with 6-recycle calculation. **(E)** Distance plot of the Cα atom per residue in Kar9^C-MIS between each two predicted models. The boundary used for local RMSD calculation is indicated by two arrowheads above the pLDDT-colored secondary structure elements.

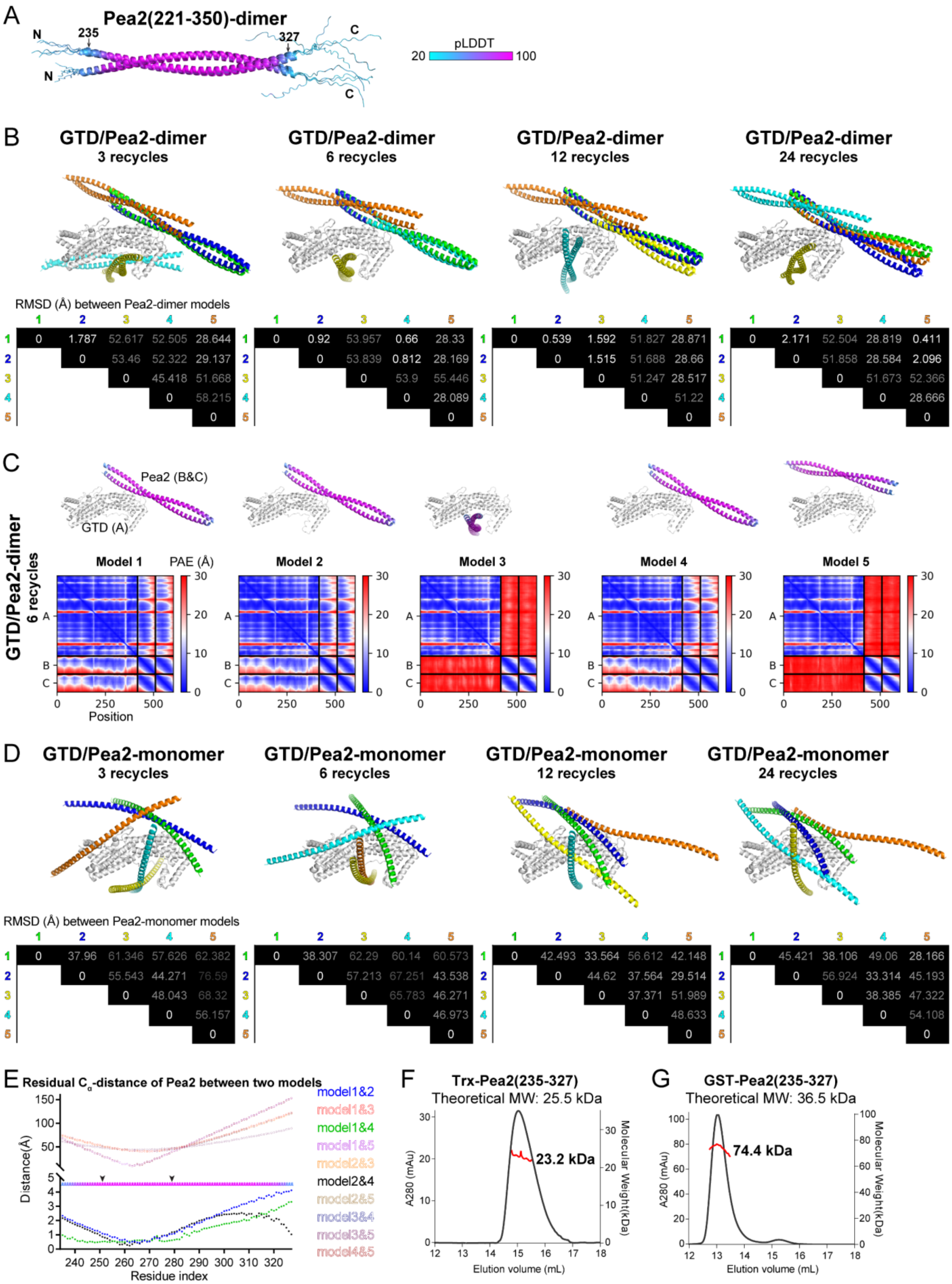

RMSD (Å) between Pea2-dimer models — 3 recycles

	1	2	3	4	5
1	0	1.787	52.617	52.505	28.644
2		0	53.46	52.322	29.137
3			0	45.418	51.668
4				0	58.215
5					0

6 recycles

	1	2	3	4	5
1	0	0.92	53.957	0.66	28.33
2		0	53.839	0.812	28.169
3			0	53.9	55.446
4				0	28.089
5					0

12 recycles

	1	2	3	4	5
1	0	0.539	1.592	51.827	28.871
2		0	1.515	51.688	28.66
3			0	51.247	28.517
4				0	51.22
5					0

24 recycles

	1	2	3	4	5
1	0	2.171	52.504	28.819	0.411
2		0	51.858	28.584	2.096
3			0	51.673	52.366
4				0	28.666
5					0

RMSD (Å) between Pea2-monomer models — 3 recycles

	1	2	3	4	5
1	0	37.96	61.346	57.626	62.382
2		0	55.543	44.271	76.59
3			0	48.043	68.32
4				0	56.157
5					0

6 recycles

	1	2	3	4	5
1	0	38.307	62.29	60.14	60.573
2		0	57.213	67.251	43.538
3			0	65.783	46.271
4				0	46.973
5					0

12 recycles

	1	2	3	4	5
1	0	42.493	33.564	56.612	42.148
2		0	44.62	37.564	29.514
3			0	37.371	51.989
4				0	48.633
5					0

24 recycles

	1	2	3	4	5
1	0	45.421	38.106	49.06	28.166
2		0	56.924	33.314	45.193
3			0	38.385	47.322
4				0	54.108
5					0

Figure S6. Structure prediction results of the Myo2-GTD in complex with Pea2.**(A)** The predicted Pea2-CC1 structure with a coiled-coil architecture. **(B)** A layout of the GTD-aligned predicted structures of Myo2-GTD in complex with the Pea2-CC1 dimer. The local RMSD values between any two predicted GTD-binding regions were calculated. **(C)** A layout of the five predicted structures and the corresponding PAE maps of Myo2-GTD in complex with the Pea2-CC1 dimer with 6-recycle calculation. **(D)** A layout of the GTD-aligned structures of Myo2-GTD in complex with the Pea2-CC1 monomer and the local RMSD values were calculated. **(E)** Distance plot of the Cα atom per residue in Pea2-1 in the Pea2-CC1 dimer between each two predicted models. The boundary used for local RMSD calculation is indicated by two arrowheads above the pLDDT-colored secondary structure elements. **(F and G)** Molecular weight measurement of Trx- **(F)** or GST-tagged **(G)** Pea2-CC1.

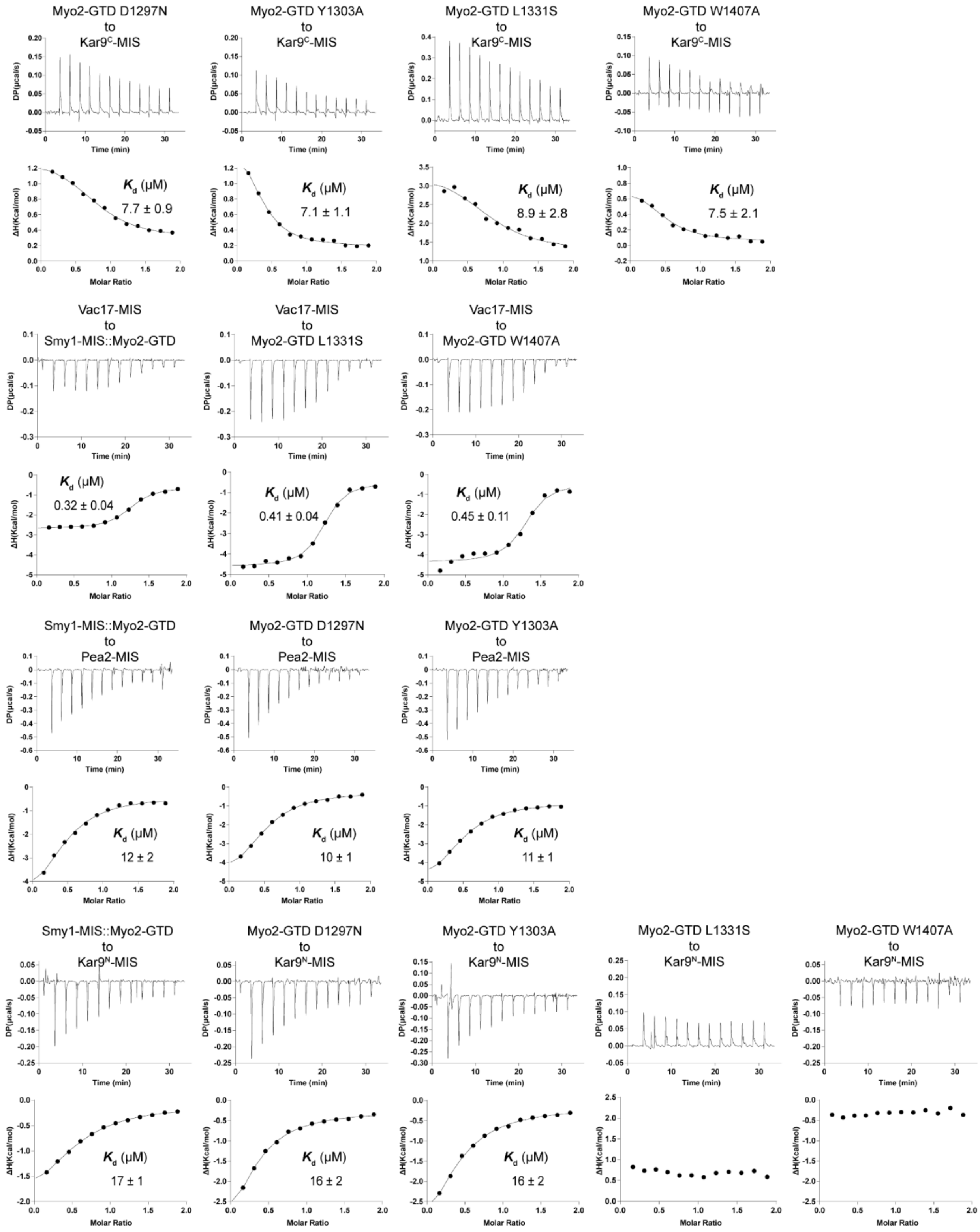

Figure S7. ITC-based analysis of Myo2-GTD or its variants binding to the MIS fragments of Kar9, Smy1, and Pea2.

Table S1. A list of the boundaries of Myo2 and the cargo adaptors used in this study.

Protein	UniProt ID	Input sequence for structure prediction	The interacting fragment for local RMSD calculation	The fragment used in ITC measurements
Myo2	P19524	1152-1574	/	1152-1574
Mmr1	Q06324	378-441	409-422	/
Smy1	P32364	578-647	637-644	/
Inp2	Q03824	504-618	534-539	/
Vac17	P25591	112-157	131-145	130-150
Kar9	P32526	1-412	/	1-412
		413-459	/	413-459
		413-530	/	413-530
		413-644	/	413-644
		531-644	/	531-644
		460-512	464-480	460-486
Pea2	P40091	221-350	/	221-350
		235-327	251-279	235-327
